# Clinical features of omicron SARS-CoV-2 variants infection associated with co-infection and ICU-acquired infection in ICU patients

**DOI:** 10.3389/fpubh.2023.1320340

**Published:** 2024-01-05

**Authors:** Dong-Jie Li, Can-Can Zhou, Fang Huang, Fu-Ming Shen, Ying-Chuan Li

**Affiliations:** ^1^Department of Pharmacy, Shanghai Tenth People’s Hospital, Shanghai, China; ^2^Department of Critical Care Medicine, Shanghai Tenth People’s Hospital, Shanghai, China

**Keywords:** COVID-19, Omicron, co-infection, ICU-acquired infection, *Candida app*

## Abstract

**Background:**

Although the decreasing rate of hospital admission in the omicron wave has led countries to loosen control, still the patients requires ICU admission. It is common for viral respiratory infections to be co-infected with bacteria. However, the difference between co-infection and ICU-acquired infection on their clinical characteristics and outcomes during the Omicron wave was little reported.

**Methods:**

Clinical and microbiological data were collected from ICU patients with omicron infection between April 1st, 2022, and May 31th, 2022 and a comprehensive comparative study of the clinical characteristics and endpoint were conducted.

**Results:**

The Omicron SARS-CoV-2 variants-infected patients requiring intensive care had high rates of co-infection (42.55%). Additionally, the ICU COVID-19 patients with co-infection showed more severe clinical features compared to those with ICU-acquired infection. Furthermore, Multivariate Cox analysis demonstrated that co-infection (hazard ratio: 4.670, *p* = 0.018) was a significant risk factor for poor outcomes in ICU patients with COVID-19. Besides, Kaplan–Meier survival curve analysis revealed that COVID-19 patients with co-infection had a significantly shorter 28-Day survival time compared to those with ICU-acquired infection (*p* < 0.001). Finally, our investigation identified a significant association between the presence of *Candida app.* in the broncho-alveolar lavage and an elevated risk of mortality (OR: 13.80, *p* = 0.002) and invasive ventilation (OR: 5.63, *p* = 0.01).

**Conclusion:**

Co-infection is prevalent among patients requiring intensive care and is linked to unfavorable outcomes in the Omicron wave. Consequently, more attention may be needed for the empirical antibacterial treatment in ICU patients within the COVID-19 Omicron variant, especially anti-fungi.

## 1 Introduction

Coronavirus disease 2019 (COVID-19) may induce the incidence of acute respiratory distress syndrome (ARDS), requiring intensive care unit (ICU) admission and invasive or noninvasive mechanical ventilation. Up to now, there are several SARS-CoV-2 variants, including Alpha, Beta, Gamma, Delta, and Omicron, the newest variant, which was first confirmed in November 2021 in South Africa (1). In comparison with the previous four variants, the Omicron variant has the highest mutation rate, with 50 mutations accumulating in its genome (2). As many countries are becoming dominated by the Omicron strain, COVID-19 prevention and control have become more challenging due to its fast-spreading property and immune escape (3). Currently, Omicron and its sub-variants caused successive outbreaks, with many countries currently experiencing their more than once wave. Importantly, although most patients in Omicron wave are asymptomatic, or symptomatic people who could heal on their own, still some patients require ICU admission. Therefore, more efforts are needed to investigate the clinical characteristics of critically ill patients with Omicron SARS-CoV-2 variants infection.

The presence of respiratory viral infections may increase the risk of serious bacterial and fungal infections, leading to increased mortality, and thus co-infection caused by respiratory pathogens including bacterial and fungal is a challenging issue in COVID-19. Chen and colleagues recorded a high co-infection with both bacterial and fungal in COVID-19 patients in 2019 (4). Lansbury et al. showed that 7% of hospitalized COVID-19 patients suffered a bacterial co-infection and 14% needed intensive care (5). In support of these, Buehler et al. also found that COVID-19 patients with bacterial pulmonary superinfection had a severer disease course, particularly a lower probability of being alive and free of invasive mechanical ventilation at study day 28 (6). However, the mortality rates from COVID-19 co-infection have varied widely from twofold compared to non-co-infected COVID-19 patients, to having no effect on mortality among co-infected ICU patients (7–9). Besides, it should be noted that all these results were obtained from patients with non-Omicron variants of SARS-CoV-2. Given these controversial results, investigation on the co-infection and ICU-acquired infection may be necessary for the clinical decision of antimicrobial treatment in COVID-19 therapy in the ICU.

In this study, we aimed to describe the demographic characteristics, hematological parameters, and pathogen infections in ICU patients with Omicron SARS-CoV-2 variants infection and comparison of clinical features of between co-infection or ICU-acquired infection.

## 2 Materials and methods

### 2.1 Study design and participants

This retrospective study was conducted in the Shanghai Municipal Center for Disease Control and Prevention, one of the institutions designated to treat SARS-CoV-2 infections. All cases were confirmed to be infected with COVID-19 by performing real-time reverse transcription PCR testing. After excluding the patients who had not provided their consent, from April 1st, 2022 to May 31th, 2022, a total of 47 COVID-19 patients needed ICU care were included in the study. Considering that COVID-19 outbreak in Shanghai during this period was attributed to the heightened contagiousness of Omicron, therefore, these cases in our study were suspected to be infected with Omicron SARS-CoV-2 variants, although virus genotyping was not conducted to identify the Omicron COVID-19 strain. The protocols in this study was approved by the Ethical Committee of the Shanghai Tenth People’s Hospital (SHYS-IEC-5.0/22 K131/P01) and has also been registered on chictr.org.cn (ChiCTR2300070486).

### 2.2 Study definition

Clinical classification at admission was based on the criteria of the Guidelines for the Diagnosis and Treatment of COVID-19 of China (9th version), and disease severities were classified as mild, moderate, severe, and critical. Co-infection was defined as bacterial/fungal infections, which were detected within the first 48 h of ICU admission. ICU-acquired infection was considered bacterial/fungal infections, which occurred >48 h after ICU admission. The definition for pathogen detection was based on at least one positive result detected in broncho-alveolar lavage, blood, or urine which were collected through the electronic patient records.

### 2.3 Clinico-pathological parameters of study participants

Demographic information, clinical details, and 28-day mortality were obtained through the electronic patient records. The following data were collected: age, gender, initial symptoms, comorbidities, laboratory tests (within the first 48-h), microbiologic results were detected by metagenomics next generation sequencing (broncho-alveolar lavage, blood, and urine), and the type of mechanical ventilation. To ensure accuracy, the data was collected and reviewed by two researchers independently.

### 2.4 Statistical analysis

Continuous and categorical variables were presented as the median interquartile range (IQR) and N (%), respectively. Chi-squared test or Fisher’s exact test was applied for categorical data, and Wilcoxon rank-sum test was applied for continuous variables between the Co-infection group and the ICU-acquired infection group. Multivariate Cox proportional-hazard analysis was used to estimate the independent prognosis factors in COVID-19 patients, and comorbidities including hypertension, diabetes, cardiovascular disease, nervous system diseases, chronic respiratory disease, and cancer were included as covariates. The Kaplan–Meier method and log-rank test were used to compare the prognosis of COVID-19 patients in two groups. Univariate logistic regression model was used to calculate the death and invasive ventilation risk associated with each isolated pathogen. For statistical difference of mean of different immunity and inflammatory markers in COVID-19 co-infection groups Krusk al-Wallis test was used. SAS software 9.4 (SAS Institute Inc., Cary, NC, USA) was adopted for the statistical analyses. *p* < 0.05 was considered significant while *p*-values between 0.05 and 0.10 was considered borderline statistically significant.

## 3 Results

### 3.1 Demographic and clinical characteristics

According to the results of pathogen detection within the first 48 h of ICU admission. All the participants included in our study were divided into the co-infection (n = 20) or the ICU-acquired infection (n = 27). The baseline characteristics of the patients are summarized in Table 1. In all the participants, 29 (61.70%) patients were males with a median age of 79.72 years (Rang: 74.00–89.00). The patients in the co-infection group were borderline significantly older than the ICU-acquired infection group (85.50 vs. 77.00, *p* = 0.061). In terms of clinical classification, 20 (42.55%) patients were severe type, and 27 (57.45%) patients were critically ill type. When we classified by co-infection or ICU-acquired infection, there was a significantly higher percentage of patients with critically ill type in the co-infection group than in the ICU-acquired infection group (75.00% vs. 44.44%, *p* = 0.037). In addition, the most common symptom at the beginning of the disease was fever (44.48%), cough (48.94%), dyspnea (44.68%), fatigue (46.81%), and anorexia (53.19%). A borderline difference on dyspnea was found between co-infection and ICU-acquired infection (60.00% vs.33.33%, *p* = 0.069).

**Table 1:** Main characteristic of older adults ICU patients with Omicron SARS-CoV-2.

Variables	Total (*N* = 47)	Co-infection (*N* = 20)	ICU-acquired infection (*N* = 27)	*p* Value
**Age (years)**	79.72 (74.00–89.00)	85.50 (76.5–90.5)	77.00 (71.0–88.0)	0.061
**Gender**				0.689
Male	29 (61.70)	13 (65.00)	16 (59.26)	
Female	18 (38.30)	7 (35.00)	11 (40.74)	
**Clinical classification**				**0.037**
Severe cases, *n* (%)	20 (42.55)	5 (25.00)	15 (55.56)	
Critically ill type, *n* (%)	27 (57.45)	15 (75.00)	12 (44.44)	
**Comorbidities**				
Hypertension	28 (59.57)	14 (70.00)	14 (51.85)	0.210
Diabetes mellitus	16 (34.04)	8 (40.00)	8 (29.63)	0.458
Cardiovascular disease	27 (57.45)	14 (70.00)	13 (48.15)	0.134
Nervous system diseases	25 (53.19)	10 (50.00)	15 (55.56)	0.706
Chronic respiratory disease	29 (61.70)	14 (70.00)	15 (55.56)	0.314
Cancer	9 (19.15)	5 (25.00)	4 (14.81)	0.380
**Initial symptoms**				
Fever	21 (44.68)	8 (40.00)	13 (48.15)	0.579
Cough	23 (48.94)	9 (45.00)	14 (51.85)	0.642
Nausea/vomiting	9 (19.15)	4 (20.00)	5 (18.52)	0.898
Dyspnea	21 (44.68)	12 (60.00)	9 (33.33)	0.069
Hemoptysis	4 (8.51)	1 (5.00)	3 (11.11)	0.458
Fatigue	22 (46.81)	11 (55.00)	11 (40.74)	0.333
Anorexia	25 (53.19)	11 (55.00)	14 (51.85)	0.831
**Mechanical ventilation**				**0.003**
Non-invasive ventilation	30 (63.83)	8 (40.00)	22 (81.48)	
Invasive ventilation	17 (36.17)	12 (60.00)	5 (18.52)	
**Length of ICU stay (days)**	16 (11, 31)	14 (7.5–26.5)	24 (14–32)	0.079
**Day-28 mortality**				**0.001**
Alive	29 (61.70)	7 (35.00)	22 (81.48)	
Death	18 (38.30)	13 (65.00)	5 (18.52)	

ICU = intensive care unit.

Data are median (interquartile range [IQR]) or n/N (%). p values were calculated by chi-squared test, Fisher’s exact test or Mann–Whitney U test, as appropriate.

Furthermore, in all the participants, 30 patients received non-invasive ventilation while 17 patients received invasive ventilation. When we classified the type of mechanical ventilation as invasive ventilation or not, there was a significant difference between co-infection and ICU-acquired infection (*p* = 0.003). The length of ICU stay for patients with co-infection was 14 days (Range, 7.5–26.5) while that for patients with ICU-acquired infection was 24 days (Range, 14–32), and further analysis revealed that there was a borderline significant difference in the length of ICU stay between these two groups (*p* = 0.079). Besides, 18 patients (38.30%) were death and 29 (61.70%) alive in day-28. Thirteen patients with the co-infection were dead (65%) while 5 patients in the ICU-acquired group (18.52%) were dead in day 28. The proportions of mortality patients were significantly different between the two groups (*p* = 0.001).

### 3.2 Hematological parameters of ICU patients with SARS-CoV-2 omicron variant and co-infection or ICU-acquired infection

Laboratory findings of the participants were described in Table 2. There were no differences in the levels of white blood cell count (12.79 [7.64–14.73] vs. 8.93 [5.72–14.91], *p* = 0.424), C reactive protein (CRP) (76.40 [40.48–132.01] vs. 49.22 [21.38–97.91], *p* = 0.254), and hemoglobin (107 [198.50–120.50] vs. 104 [84–129], *p* = 0.739) between the co-infection and ICU-acquired infection groups. However, that the absolute numbers of lymphocytes count (0.49 [0.28–0.69] vs. 0.88 [0.60–1.05], *p* = 0.006) were significantly lower in co-infection patients than that in ICU-acquired infection patients. Compared with the ICU-acquired infection, the co-infection patients had significantly higher AST (37.50 [30.50–58.50] vs. 28 [20–45], *p* = 0.044) and serum urea (10.29 [8.26–19.30] vs. 6.45 [5.30–10.00], *p* = 0.032) levels. Meanwhile, serum ALP levels (96 [76–133] vs. 88 [65–95], *p* = 0.099) and creatinine levels (92.95 [64.60–161.25] vs. 61.10 [49.10–103.90], *p* = 0.085) also tended to be higher in the co-infection group with a borderline significance.

**Table 2:** Laboratory findings of patients with SARS-CoV-2 Omicron infection at admission.

Parameters	Normal Range	Total (*N* = 47)	Co-infection (*N* = 20)	ICU-acquired infection (*N* = 27)	*p* Value
**Blood routine examination**					
White blood cells,x10^9^/L	3.5–9.5	10.38 (6.10–14.89)	12.79 (7.64–14.73)	8.93 (5.72–14.91)	0.424
Lymphocytes,x10^9^/L	1.1–3.2	0.77 (0.39–1.01)	0.49 (0.28–0.69)	0.88 (0.60–1.05)	**0.006**
Platelets,x10^9^/L	125–350	180 (145–248)	169.50 (108.50–212)	193.50 (158–249)	0.263
Hemoglobin, g/L	115–150	106 (88–127)	107 (198.50–120.50)	104 (84–129)	0.739
**Biochemical examination**					
C-reactive protein, mg/L	0–10	52.55 (25.80–111.07)	76.40 (40.48–132.01)	49.22 (21.38–97.91)	0.254
Alanine aminotransferase, U/L	7–40	19.00 (13.00–46.00)	20.50 (17.50–37.50)	19.00 (13.00–46.00)	0.334
Aspartate aminotransferase, U/L	13–35	32 (21–49)	37.50 (30.50–58.50)	28 (20–45)	**0.044**
Total bilirubin, μmol/L	3.4–20.5	13.95 (9.40–21.00)	14.10 (8.90–26.10)	13.05 (10.50–19.40)	0.595
ALP, U/L	50–135	91.50 (69–112)	96 (76–133)	88 (65–95)	0.099
Creatinine, μmol/L	41–81	75.10 (52.60–120.00)	92.95 (64.60–161.25)	61.10 (49.10–103.90)	0.085
Uric acid, μmol/L	150–350	308.02 (168.96–421.65)	369.43 (176.75–468.96)	234.32 (168.96–375.51)	0.177
Urea, mmol/L	3.1–8.8	8.97 (5.89–13.30)	10.29 (8.26–19.30)	6.45 (5.30–10.00)	**0.032**
Lactate dehydrogenase, U/L	120–250	332.5(254–391)	351.50 (257–473.50)	324 (253–366)	0.308
K^+^, mmol/L	3.5–5.3	3.90(3.40–4.26)	3.95(3.45–4.23)	3.9(3.40–4.30)	0.534
Na^+^, mmol/L	137–147	139(135–144)	141(136–144)	139(134–143)	0.262
**Coagulation function**					
Prothrombin time, sec	11–14.5	14.95 (13.50–16.50)	15.40 (13.90–16.65)	14.90 (13.20–16.50)	0.425
Fibrinogen, g/L	2.0–4.0	4.68 (3.63–5.71)	4.82 (3.18–6.17)	4.57 (3.64–5.20)	0.407
D-dimer, μg/mL	0–0.50	2.50 (1.32–6.03)	2.15 (1.03–6.65)	2.81 (1.42–6.03)	0.623
**Immune/inflammatory factors**					
CD3^+^, cell/μL	690–2,540	339.02 (161.94–706.30)	231.47 (136.87–413.29)	631.62 (279.85–711.24)	**0.032**
CD4+, cell/μL	410–1,590	231.11 (107.23–402.39)	190.53 (102.01–293.85)	256.83 (195.59–419.52)	0.232
CD8+, cell/μL	190–1,140	75.98 (41.08–286.27)	54.49 (22.28–77.60)	158.17 (67.10–321.33)	**0.008**
CD4/CD8 ratio	0.90–3.60	2.51 (1.31–4.49)	3.76 (2.73–5.41)	1.51 (1.19–2.75)	**0.004**
TNF-α, pg./mL	< 16.50	0.34 (0.13–1.70)	0.23 (0.13–1.81)	0.34 (0.06–1.70)	0.728
IL-6, pg./mL	< 5.40	6.29 (0.00–29.64)	9.58 (6.02–54.61)	0.00 (0.00–29.64)	**0.022**
IL-10, pg./mL	< 12.90	13.83 (8.55–144.76)	34.37 (12.79–693.04)	9.86 (2.79–44.64)	**0.034**

ICU = intensive care unit; ALP = alkaline phosphatase; K+ = Potassium ion; Na+ = Sodium ion; TNF-α = Tumor necrosis factor-α; IL-6 = Interleukin-6; IL-10 = Interleukin-10.

Data are median (interquartile range [IQR]) or n/N (%). *p* values were calculated by chi-squared test, Fisher’s exact test or Mann–Whitney U test, as appropriate.

Considering that immunity and inflammatory responses are closely associated with the clinical manifestations of COVID-19 and outcomes (10, 11), the levels of peripheral CD3^+^, CD4^+^, and CD8^+^ T cells as well as the levels of TNF-α, IL-6, and IL-10 were also determined in this study. Compared with ICU-acquired infection patients, co-infection groups had lower levels of CD3^+^ T cells (231.47 [136.87–413.29] vs. 631.62 [279.85–711.24], *p* = 0.014). No significant differences observed in CD4+ cells, but co-infection patients had lower levels of CD8^+^ T cells (54.49 [22.28–77.60] vs. 158.17 [67.10–321.33], *p* = 0.008), and higher CD4/CD8 ratio (3.76 [2.73–5.41] vs. 1.51 [1.19–2.75], *p* = 0.004) than that of ICU-acquired infection patients. Furthermore, compared with ICU-acquired infection patients, co-infection group had a significantly higher levels of serum IL-6 (9.58 [6.02–54.61] vs. 0.00 [0.00–29.64], *p* = 0.022) and IL-10 (34.37 [12.79–693.04] vs. 9.86 [2.79–44.64], *p* = 0.034) (Table 2). Collectively, all these laboratory tests indicated that ICU patients with co-infection had severer inflammatory responses as well as worse liver and kidney function index compared with those with ICU-acquired infection.

### 3.3 Identification of pathogen infections In omicron patients with ICU care

**Figure 1:**
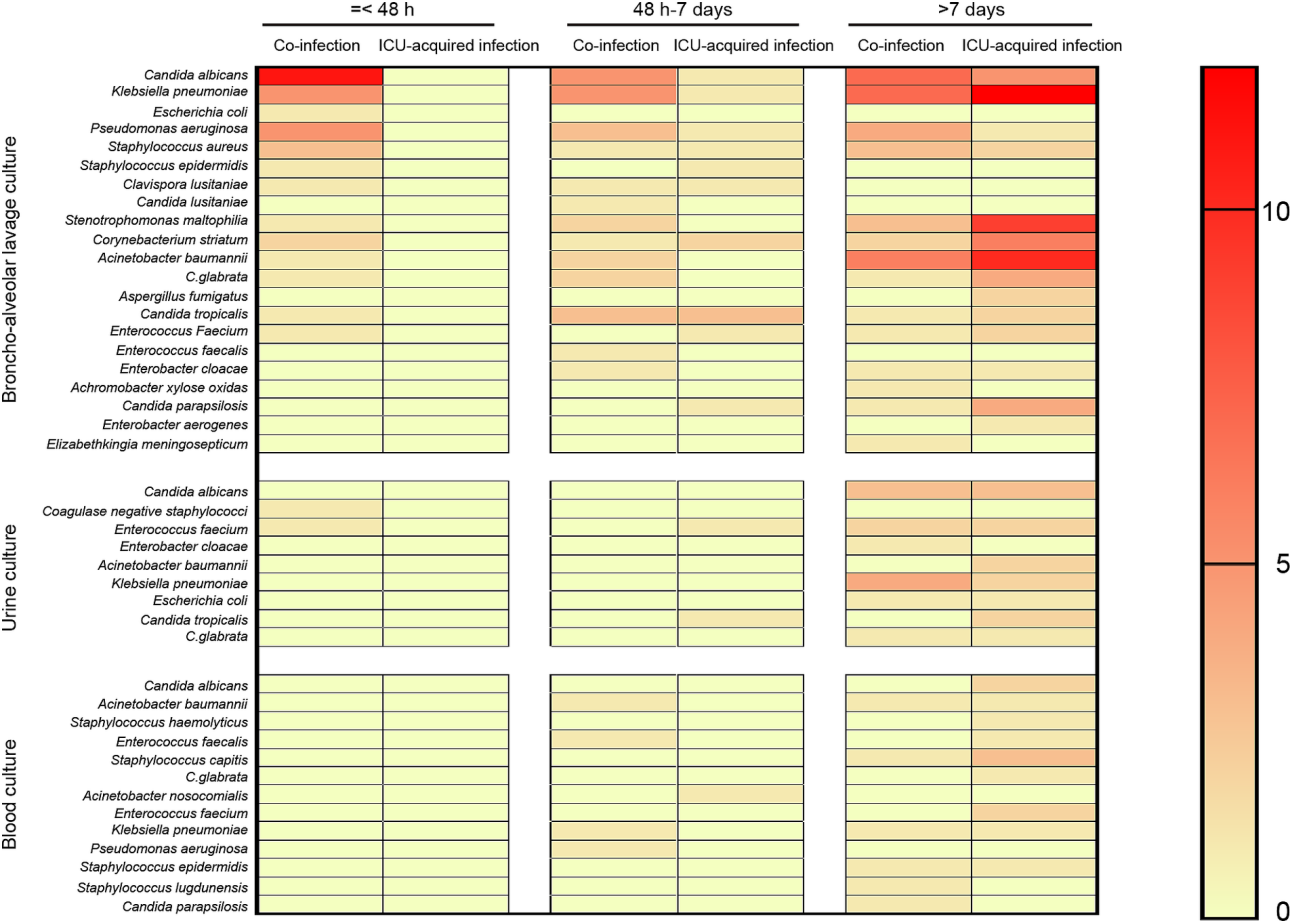
Distribution of infection pathogens positive results stratified by days. The potential pathogens were detected by metagenomics next generation sequencing (broncho-alveolar lavage, blood, and urine) and stratified by days (≤ 48 h, 48 h − 7 days, and ≥ 7 days) following ICU admission.

The list of identified potential pathogens by metagenomics next generation sequencing (broncho-alveolar lavage, blood, and urine) in co-infection and ICU-acquired infection patients stratified by days following ICU admission were shown in Figure 1. Fifteen potential pathogens were identified from 20 patients in the co-infection group within the first 48 h of ICU admission. And the most common co-pathogen in broncho-alveolar lavage were *Candida albicans* (n = 11), *Klebsiella pneumonia* (n = 5), *Pseudomonas aeruginosa* (n = 5), and *Staphylococcus aureus* (n = 3) and 11 patients had more than one coinfected bacterium. Furthermore, two patients were also detected to be infected with *Coagulase-negative staphylococci* and *Enterococcus Faecium* in urine, respectively.

Beyond 48 h of hospital admission to the end of ICU stay, 21 potential co-pathogens were identified in broncho-alveolar lavage, 8 potential co-pathogens were identified in urine, and 13 potential co-pathogens were identified in blood. When we stratified by days, 16, 2, and 5 potential pathogens were identified in broncho-alveolar lavage, urine, and blood, respectively from days 3–7 and 17, 8, 11 potential pathogens were identified in broncho-alveolar lavage, urine, and blood, respectively from day 8 onwards (Figure 1).

### 3.4 Co-infection Is associated with poor outcomes In ICU patients with omicron variants infection

Results of multivariate analysis on the risk of severe clinical events in these 47 patients are shown in Table 3. In the multivariate-adjusted Cox proportional hazards model, co-infection was observed to be a meaningful factor associated with the occurrence of outcomes of the patients. The ICU patients with co-infection (HR = 4.670, 95% CI: 1.298–16.802, *p* = 0.018) had a significant increased risk of day-28 mortality.

**Table 3:** Multivariate analysis for the risk of severe events in older adults ICU patients with SARS-CoV-2 Omicron.

Clinical factors	Day-28 mortality			Mechanical ventilation		
	HR	95% CI	*p* Value	HR	95% CI	*p* Value
Age	1.050	0.990–1.114	0.106	1.044	0.986–1.105	0.144
Sex	0.787	0.247–2.505	0.685	0.966	0.268–3.487	0.958
Hypertension	1.419	0.432–4.662	0.564	0.555	0.161–1.910	0.350
Diabetes	0.633	0.204–1.964	0.429	0.638	0.184–2.213	0.478
**Co-infection**	**4.670**	**1.298–16.802**	**0.018**	**5.715**	**1.553–21.035**	**0.009**
Cardiovascular diseases	1.141	0.371–3.503	0.818	1.108	0.332–3.697	0.867
Chronic respiratory diseases	0.790	0.233–2.682	0.705	0.754	0.206–2.760	0.669
Nervous system diseases	2.141	0.544–8.429	0.276	1.372	0.384–4.900	0.627
Cancer	1.660	0.362–7.611	0.514	0.673	1.053–2.950	0.599

HR = hazard ratio; CI = confidence interval.

**Figure 2:**
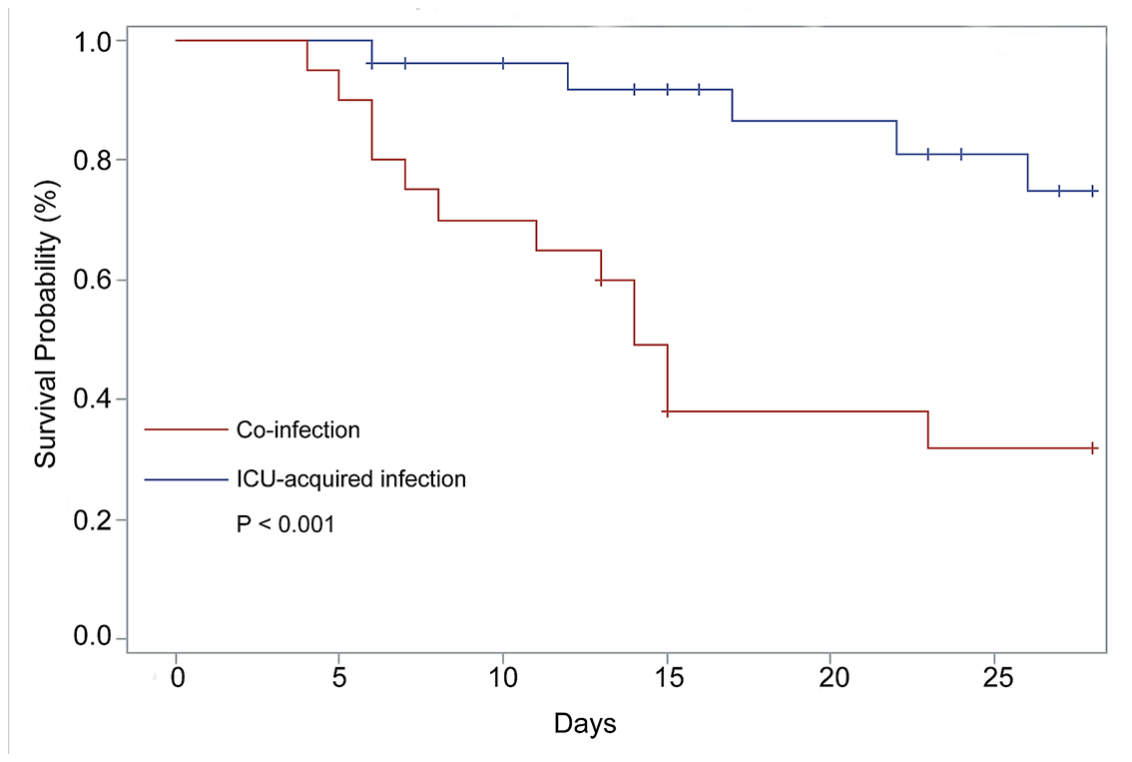
Comparison of 28-Day survival of the COVID-19 patients need ICU care with co-infection and ICU-acquired infection. The data from 47 patients contributed to these analyses. N = 20, co-infection; N = 27, ICU-acquired infection.

Furthermore, risk factors for the type of mechanical ventilation were also assessed. We found that the older adults ICU patients with co-infection (HR = 5.715, 95%CI: 1.553–21.035, *p* = 0.009) also had a significantly increased risk of invasive ventilation. The survival curve for clinical outcomes based on co-infection shown in Figure 2 indicates that the patients with co-infection had poor outcomes compared with those with ICU-acquired infection (*p* < 0.001).

### 3.5 *Candida* spp. Is a significant risk factor for death In ICU patients with omicron variants infection

**Figure 3:**
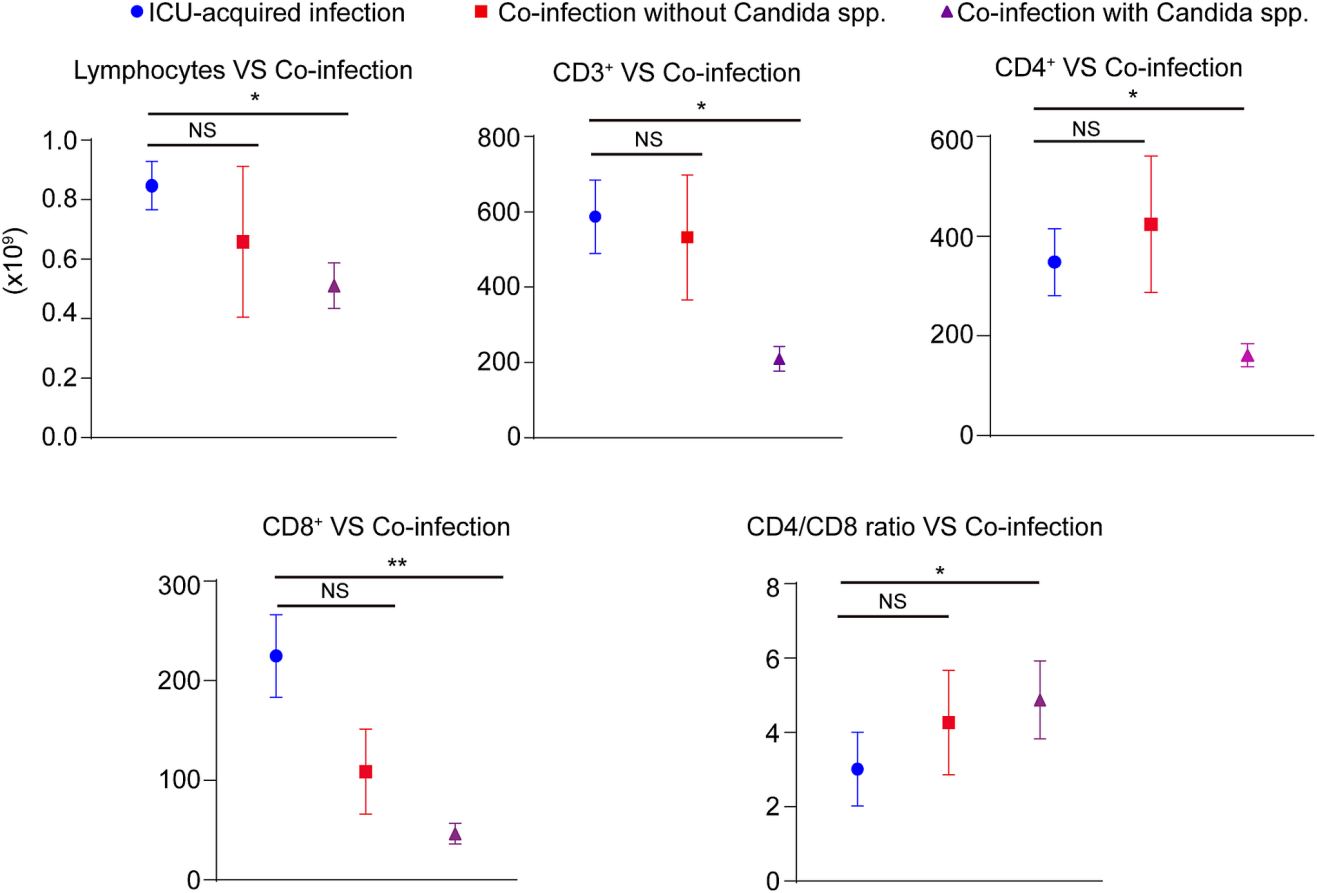
The role of immunity and inflammatory markers in COVID-19 patients. The absolute numbers of lymphocytes count as well as the levels of peripheral CD3^+^, CD4^+^, and CD8^+^ T cells were obtained through the electronic patient records. Stratifing the COVID-19 patients as ICU-acquired infection, co-infection patients with *Candida* spp. infection, and co-infection without *Candida* spp. infection. NS; NO significant, *p < 0.05, ^**^p < 0.01.

In most co-infections, more than one micro-organism was isolated from broncho-alveolar lavage. The results showed that the presence of *Candida* spp. in the broncho-alveolar lavage was associated with an increased risk of death (OR: 13.80, *p* = 0.002) and invasive ventilation (comparing with co-infection patients who not infected with *Candida* spp.) (OR: 5.63, *p* = 0.01). However, these associations were not observed when *Klebsiella* spp., *Staphylococcus* spp., *Acinetobacter* spp., or *Pseudomonas* spp. existed in the broncho-alveolar lavage (Table 4). Furthermore, when we stratified the co-infection patients as co-infection with or without *Candida* spp., we discovered that compared with ICU-acquired infection, the patients co-infected with *Candida* spp. showed significantly lower lymphocyte numbers, CD3^+^, CD4^+^, and CD8^+^ T cells, along with a higher ratio of CD4/CD8. Conversely, no differences were observed between ICU-acquired infection and co-infection without *Candida* spp. (Figure 3).

**Table 4:** Risk of death associated with isolation of bacteria and fungi from COVID-19 patients.

Genus	Day-28 mortality		Mechanical ventilation	
	OR (95% CI)	*P*-value	OR (95% CI)	*P*-value
*Candida* spp.	13.80 (2.60, 7.28)	0.002	5.63 (1.46, 5.75)	0.01
*Klebsiella* spp.	Undefined^a^	0.967	1.20 (0.18, 8.00)	0.851
*Staphylococcus* spp.	Undefined^a^	0.963	0.87 (0.14, 5.31)	0.877
*Acinetobacter* spp.	Undefined^a^	0.967	8.92 (0.91, 8.84)	0.061
*Pseudomonas* spp.	5.33 (0.55, 5.88)	0.149	2.99 (0.45, 2.06)	0.257

a Undefined because odds ratio (OR) could not be calculated with a zero cell. OR and 95% confidence interval (CI) were calculated using univariate logistic regression model. Data were calculated considering the isolated micro-organisms individually.

## 4 Discussion

According to our knowledge, this is the first study to be conducted in China on the comparison of clinical features of associated with co-infection and ICU-acquired infection in ICU patients following Omicron variant infection. This work describes four major novel findings. First, our results showed that the COVID-19 patients need ICU care in the Omicron wave had high rates of co-infection. Second, we found that the ICU patients with co-infection had more seriously clinical features and higher day-28 mortality compared with those with ICU-acquired infection. Thirdly, we observed that co-infection was an independent meaningful factor associated with the occurrence of invasive ventilation and day-28 mortality in ICU patients following Omicron variant infection. Finally, we found *Candida app*. in the broncho-alveolar lavage was significantly associated with an increased risk of death in ICU COVID-19 patients. Collectively, our data suggested that co-infection is common in ICU patients and associated with poor outcomes in the Omicron wave. More attention may be needed for the empirical antibacterial treatment in ICU patients within the COVID-19 Omicron variant, especially anti-fungal.

Several studies have investigated the effects of co-infection on their clinical characteristics and outcomes in COVID-19 patients. For example, Garcia-Vidal and colleagues found that compared with COVID -19 patients without infection, both hospital-acquired super infection and community-acquired co-infection patients had worse outcomes (12). Baskaran et al. also indicated that patients with co-infections were more likely to die in ICU compared to those without co-infections (13). However, few studies have elucidated the contrasting clinical characteristics and outcomes in patients with co-infection and ICU-acquired infection, especially with Omicron SARS-CoV-2 infection. As we know, the advanced age has been regarded as one of the main COVID-19 risk factors for co-infection and age-related immune system senescence is considered to be the major reason for increased susceptibility to infection (14, 15). Interestingly, in our study, compared with the ICU-acquired infection group, the elders with omicron infection in the co-infection group were associated with higher age. Furthermore, the co-infection patients displayed more symptoms of dyspnea, worse clinical classification at admission, and a higher percentage of individuals using invasive ventilation. All these clinical features suggested that co-infected patients were more likely to develop a severe condition. Besides, at the follow-up endpoint, we compared the length of ICU stay and day-28 mortality in two groups. We observed that there was a borderline difference in the length of ICU stay between the co-infection and ICU-acquired infection patients. The short length of stay in co-infected patients indicated that the co-infected older adults with omicron infection might present considerably fast disease progression. Moreover, co-infections resulted in 13 patients’ death representing a 65% mortality rate on the day-28, which was significantly higher than ICU-acquired infections. Our data showed that there was a higher percentage of patients in the co-infection group (60%) than in the ICU-acquired infection group (18.52%) treated by invasive ventilation (*p* = 0.003). Therefore, our study highlighted that although the virulence of the omicron variant is reduced, it still has a poor prognosis in the older adults, especially with co-infection.

Viral infection triggers an innate immune response and immunopathology is the main mechanism in the genesis and progression of COVID-19 (16). As we know, CD3^+^ and CD8^+^ T cells are crucial in T-cell antigen recognition and resistance to viruses or other stimuli (11). Previously, it has been reported that CD3^+^ and CD4^+^ T cell count were independent prognostic factors for death in older adults with severe community-acquired pneumonia and sepsis (17, 18). Recently, Wan et al. and Liu et al. showed that the counts of CD8+ cells were remarkably decreased in severe and critical patients with COVID-19 (19, 20). In addition, several studies showed that patients with critical or severe COVID-19 presented low CD3^+^ T cell count (21, 22). Besides, the increased higher ratio of CD4/CD8 was associated with the inflammatory status of COVID-19 (23). The CD4/CD8 ratio was considered a marker for the early identification of those likely to require intervention in the ICU (24). These reports revealed that the reduced lymphocyte count was related to the occurrence of critical COVID-19 cases. In the present study, the results showed that co-infection patients had lower levels of lymphocyte, CD3^+^, and CD8^+^ T cells, and a higher ratio of CD4/CD8 in comparison with ICU-acquired infection patients, which may contribute to the severer symptoms and worse clinical outcomes in co-infection patients.

The excessive release of cytokines and chemokines in COVID-19 results in a cytokine storm ensues (25, 26). Cytokine storm refers to a pathological state characterized by uncontrolled systemic inflammation, which is caused by the excessive cytokines production, leading to multi-organ failure and even death (27). Several cytokines, such as IL-1β, IL-6, IL-8, IL-10, TNF-α, interferon (IFN)-γ, play crucial roles in the pathogenesis of cytokine storm (28). Previous studies have highlighted the relationship between cytokine storm and severity of COVID-19, and cytokine storm is verified as a significant contributor to mortality associated with the disease (29). Prevention and mitigation of the cytokine storm may be a promising strategy to save patients with severe COVID-19 (29). Infections and tissue injuries are defended against by IL-6. However, the overproduction of IL-6 when fighting against SARS-CoV-2 may result in systemic inflammatory responses (30). There is increasing evidence indicating that IL-6 is an early biomarker of lung damage and is closely associated with prolonged mechanical ventilation, increased morbidity, and mortality in lung diseases (31, 32). For example, the increased IL-6 levels in serum and brochoalveolar lavage fluid have been found in asthmatic patients (33, 34). Huang et al. Have found that IL-6 is a strong predictor of the frequency of chronic obstructive pulmonary disease exacerbation within 1 year (35). In addition, the essential role of IL-6 in sepsis-induced acute lung injury and pulmonary arterial hypertension has also been observed (36, 37). IL-10 is highly abundant in influenza infection, especially during the adaptive immune response (37). Patients in the ICU with COVID-19 have higher peripheral IL-10 levels than those in non-ICUs (38). Furthermore, targeting IL-6 and IL-10 has been proposed for treating ARDS in COVID-19 patients based on its immunoregulatory functions (39). However, the difference between co-infection and ICU-acquired infection on the inflammatory response in ICU COVID-19 patients with omicron infection is still unclear. In addition, we found that the ICU patients with co-infection had significantly higher levels of IL-6 and IL-10 than those with ICU-acquired infection. These results are apparently in line with previous reports suggesting IL-6 and IL-10 are disease severity predictors (25, 40). Besides, co-infections also cause hematological and biochemical imbalance, worsening the general clinical condition. These results indicated that the patients with co-infection had severer inflammatory responses, which might contribute to the worse prognosis of Omicron infection.

It is common for viral respiratory infections to be co-infected with bacteria. They are the main causes of difficult diagnosis, poor outcomes, increasing morbidity and mortality, and greater healthcare costs (41). There are many pathogens that may cause respiratory co-infections, including bacteria, viruses, fungi, etc. In the current study, the most common bacteria in broncho-alveolar lavage in the older COVID-19 patients within 48 h were *Klebsiella pneumonia*, *Pseudomonas aeruginosa*, and *Staphylococcus aureus*. The predominant late pathogens observed in the broncho-alveolar lavage were *Klebsiella pneumonia*, *Stenotrophomonas maltophilia*, and *Corynebacterium striatum*. Consistent with previous results, these pathogens commonly caused hospital- and ventilator-acquired pneumonia, especially in ICU. Bacterial infections are indeed more prevalent in critically ill patients, but fungal infections are also deadly. Our data showed that in both co-infections and ICU-acquired infections, *Candida* spp. was detected with a high prevalence. As we know, *Candida* spp. is a part of the human microbiota, and it is difficult to differentiate infection from colonization. Therefore, this might be one cause for the higher rate of co-infections in our study. In line with our results, Silva et al. also reported a high prevalence of *Candida* spp., which was the main fungus causing infections in critically ill patients (42). It is noteworthy that interventions for patients with COVID-19 in ICU increased the opportunity of infections, including corticosteroids, broadspectrum antibacterial, and mechanical ventilation. Therefore, the increased risk of death may indicate that *Candida* spp. might act as the pathogen, which should not be ignored. To ascertain the essential role of co-infection on the progression of COVID-19, the Kaplan–Meier analysis and survival curves were conducted, and the results indicated that the COVID-19 patients with bacterial/fungal co-infection had a significantly poorer survival rate than ICU-acquired infection. Furthermore, multivariate Cox analysis also showed that co-infection was an independent meaningful factor associated with the occurrence of severe events, including 28-day mortality and the type of mechanical ventilation. Interestingly, further analysis showed that the isolation of *Candida* spp., but not *Klebsiella* spp., *Staphylococcus* spp., *Acinetobacter* spp., or *Pseudomonas* spp. in the broncho-alveolar lavage was associated with an increased risk of death and invasive ventilation. Besides, the patients co-infected with *Candida* spp. showed the significant changes of immunity and inflammatory markers, including lymphocyte numbers, CD3^+^, CD4^+^, CD8^+^ T cells, and the ratio of CD4/CD8.

Our study has several limitations. First, our study was retrospectively designed and the effects of confounding factors might be underestimated, therefore, more prospective studies are required. Second, it’s should be considered that the analysis cannot meet the requirements of the event per cariable due to the small sample size. However, considering the rarity of such a population and the interpretability of the results, they are still shown. Therefore, studies with a larger sample size should be carried out to validate our conclusions. Finally, our analyses were limited to the available data extracted from the electronic medical records. However, data from our study contributes to a better understanding of co-infections in patients with COVID-19 in ICU patients with the SARS-CoV-2 omicron variant.

In conclusion, our results showed that co-infection is common in ICU patients with Omicron SARS-CoV-2 infection, which displayed worse clinical features and outcomes. Importantly, *Candida app*. in the broncho-alveolar lavage was significantly associated with an increased risk of death in ICU COVID-19 patients. Collectively, our data suggested that if a ICU patient with Omicron SARS-CoV-2 infection shows strong evidence of a fungal co-infection, this possibility should not be ignored.

## Data availability statement

The original contributions presented in the study are included in the article/supplementary material, further inquiries can be directed to the corresponding author.

## Ethics statement

The studies involving humans were approved by the Ethical Committee of the Shanghai Tenth People's Hospital. The studies were conducted in accordance with the local legislation and institutional requirements. The ethics committee/institutional review board waived the requirement of written informed consent for participation from the participants or the participants' legal guardians/next of kin because this is a retrospective study and the demographic information, clinical details, and 28-day mortality were obtained through the electronic patient records.

## Author contributions

D-JL: Conceptualization, Resources, Writing – original draft. C-CZ: Data curation, Writing – original draft. FH: Data curation, Writing – review & editing. F-MS: Conceptualization, Writing – review & editing. Y-CL: Conceptualization, Investigation, Resources, Writing – review & editing.
